# Effects of Different Methods of Air Disinfection of Computed Tomography Rooms Dedicated to COVID-19 Cases

**DOI:** 10.1155/2020/5302910

**Published:** 2020-11-25

**Authors:** Yilian Cheng, Jing Hu, Hui Chen, Liu Wu, Jianmei Liao, Lin Cheng

**Affiliations:** ^1^Department of Radiology, Southwest Hospital, Third Millitary Medical University (Army Medical University), Chongqing, 400038, China; ^2^Infection Control Department, Southwest Hospital, Third Millitary Medical University (Army Medical University), Chongqing, 400038, China; ^3^Nursing Department, Southwest Hospital, Third Millitary Medical University (Army Medical University), Chongqing, 400038, China

## Abstract

**Objective:**

To monitor the number of bacterial colonies in the air of computed tomography (CT) room for COVID-19 using different disinfection methods and to identify the most effective method for disinfection and protection of equipment.

**Methods:**

Three methods for disinfection using ultraviolet germicidal irradiation (group A), plasma circulation air sterilizer (group B), and ultraviolet germicidal irradiation plus plasma circulation air sterilizer (group C) were utilized to sanitize the air in the CT room dedicated to COVID-19 cases. Single-factor ANOVA was used to evaluate and compare the disinfection effect of the three air disinfection methods; an air microbial sampler was used to sample and measure the number of bacteria in the air of the machine room.

**Results:**

The number of bacteria in the air immediately after disinfection was significantly lower than before disinfection (*p* < 0.01). All three disinfection methods met the disinfection requirement. No significant differences in the number of air bacteria in the machine room immediately after disinfection were observed among the three methods (*p* > 0.05). In addition, the effect of disinfection after 2 h was compared, and the number of bacteria in group C after 2 h was significantly lower than that in group A and group B.

**Conclusions:**

All three disinfection methods have significant disinfection effects. In addition, using ultraviolet disinfection lamps combined with a plasma air disinfection machine to sterilize the air in CT machine room has the best disinfection effect for the longest duration. Therefore, we recommend the combined disinfection method (ultraviolet disinfection lamps plus plasma air disinfection), as well as formulating relevant disinfection management norms, which should thus be the method to use during pandemics.

## 1. Introduction

In December 2019, multiple unusual viral pneumonia cases were reported in Wuhan City, Hubei Province, China, and immediately followed by further spread to more than 30 cities and provinces across the country [1]. By January 12, 2020, the virus involved in this unusual viral pneumonia outbreak was identified as a novel coronavirus belonging to the genus *Betacoronavirus* [2]. It was then named as severe acute respiratory syndrome coronavirus 2 (SARS-CoV-2) by the International Committee on Taxonomy of Viruses. Thereafter, the World Organization of Health officially designated the disease caused by SARS-CoV-2 infection as coronavirus disease 2019 (COVID-19) [3]. Further investigations have revealed essential viral characteristics such as multiple transmission routes, long survival time, and general susceptibilities [4]. Organized disinfection and isolation have also been established as important measures for the prevention of further spread of the virus [5].

Pulmonary computed tomography (CT) scan is one of the most representative and accurate diagnostic methods for COVID-19 [6]. The radiology department, together with the outpatient clinic of general fever, has designated specific CT rooms for COVID-19 and established a strict disinfection and isolation system to avoid cross infection in most hospitals in China. According to the *Guidelines for the Diagnosis and Treatment of the Novel Coronavirus Pneumonia* (Trial edition 5) issued by the National Health Commission of the People's Republic of China, air disinfection in medical institutions during epidemics may be any of the following disinfectants: chlorine disinfectant, peracetic acid, chlorine dioxide, and hydrogen peroxide, and the ultralow volume spray method should be used [7]. In addition, ultraviolet (UV) irradiation has been recommended by other reports for disinfection because SARS-CoV-2 is sensitive to UV light and is deactivated within 30 min of irradiation [8]. However, in view of the structure and the particularity of the machinery and equipment of the CT room, both recommended disinfection methods still have limitations [9].

This study is based on the recommendations of the *Guidelines for the Diagnosis and Treatment of the Novel Coronavirus Pneumonia* and other related literature and combined with the daily disinfection management of the CT room [10–12]. We employed different methods for air disinfection to compare and analyze the disinfection effects and safety of the designated CT room, thereby selecting the most effective disinfection method. This study provides the basis for the disinfection of machinery and equipment during a COVID-19 epidemic.

## 2. Materials and Methods

### 2.1. Tested Objects

The number of bacterial colonies in the air of the room containing a 128-slice CT scanner (Model: Definition, German Siemens Company), with an area of approximately 45 square meters, was the tested object in this study.

### 2.2. Instrument Equipment

The parameters of the instrument equipment in this study were listed as follows: UV disinfection lamps (Model: ZW30S19W (Y), Jiang Su Juguang Photoelectric Co. LTD, China), plasma circulation air sterilizer (Model: SKW-DJX-G150, Chengdu San Kangwang Disinfection Equipment Co. LTD, China), microbial air sampler (Model: MAS-100 NTTM, Merck KGaA), and CO2 constant temperature incubator (Model: 3111, Thermo Scientific Inc).

### 2.3. Methods for Disinfection

Three methods for disinfection using UV germicidal irradiation (group A), plasma circulation air sterilizer (group B), and UV germicidal irradiation plus plasma circulation air sterilizer (group C) were utilized to air sanitize the CT room that is dedicated to COVID-19. Each group collected air samples for 6 days at 5 time points, using three replicates for each time point each day. Each group obtained 24 sample data.

In group A, 4 straight tube UV disinfection lamps of 30 W were installed in the CT room according to the room size, with the lamp placed approximately 2 m from the ground. At the end of CT examination and when nobody was inside the CT room, UV germicidal irradiation was performed every 8 h by turning on the UV lamp for 60 min (with the timer starting 5–7 min after turning on the lamp); then, the lamp was turned off [13], and 10 min later, medical personnel entered the CT room for sampling.

In group B, a plasma air sterilizer was used for air disinfection by turning on the sterilizer every 8 h at the end of CT examination and when nobody was inside the CT room, with an average circulating air volume of ≥1,000 m^3^/h. The sterilizer was turned off after 60 min of disinfection [14], and 10 min later, medical personnel entered the CT room for sampling.

In group C, at the end of the inspection, when there is no one in the room, the plasma air sterilizer (average circulating air volume ≥ 1,000 m^3^/h) and the ultraviolet disinfection lamps (the timer started after the light was on for 5–7 min) were turned on at the same time every 8 h. Approximately 10 min later, medical personnel entered the CT room for sampling.

### 2.4. Sample Collection

Sample collection was performed 10 min after the 60 min air disinfection and thrice a day.

For sample collection, 90 mm nutrient agar plates were placed into the microbial sampler to collect air samples from the machine room using the five-point positioning method at the sampling flow rate of 100 L/min, and then, samples were collected from each sampling point for 1 min. After sampling, the nutrient agar plates were placed in a 37°C incubator for 48 h; then, the number of colonies and total number of bacteria were counted [15].

### 2.5. Statistical Analysis

This study used the SPSS22.0 software (IBM SPSS Inc., Chicago, IL, USA) for data analysis. The measurement data were presented as the mean ± standard deviation. The quantile-quantile graph in SPSS22.0 was used to test the normality of the data, and single-factor ANOVA was used to detect the differences among multiple groups in this study. Differences with *p* < 0.05 were considered statistically significant.

## 3. Results

**Figure 1:**
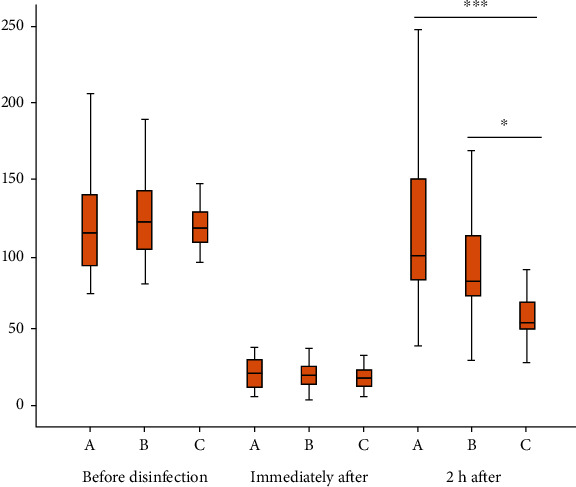
The significant different disinfection effects of these three disinfection methods at three time points.

There was no significant difference in the number of air bacteria in the machine room before the three disinfection methods were used (*p* > 0.05). Compared with the three methods before disinfection, the number of air colonies after disinfection was significantly reduced (*p* > 0.01, Table 1). The comparison between groups showed that there was no significant difference in the disinfection effect immediately after disinfection by the three methods (*p* > 0.05), whereas at 2 h after disinfection, the number of bacterial colonies of group C was significantly lower than that of groups A and B (*p* > 0.01, Figure 1, Table 2).

## 4. Discussion

We found the characters of chest CT were important in COVID-19 diagnosis which could significantly distinct COVID-19 from common influenza [16] and also had several advantages, such as high sensitivity, relative characteristics, convenience, noninvasive, and rapid.

The main transmission route of SARS-CoV-2 is droplet transmission and contact transmission; aerosol transmission has also been found. Paying attention to disinfection management of the hospital environment is the most important prevention and control method. The disinfection strategies should established in examination room, especially in CT room, according to its specialty and layout. In this study, we designed several different disinfection methods according to the Technical Specification for Disinfection in Medical Institutions [17], *Guidelines for the Diagnosis and Treatment of the Novel Coronavirus Pneumonia* (Trial edition 5), and the disinfection equipment in our department and investigated their disinfection effects.

Disinfectant spraying has been the most extensively used method for air disinfection, generally spraying 500 mg/L of chlorine-based disinfectant at 10 mL/m^3^. However, the CT equipment is composed of a variety of sophisticated electronic components and must be kept in a room with the temperature and humidity maintained at a relatively constant range of 18–22°C and 40–60%, respectively [18], which are altered by disinfectant spraying. In addition, liquid diffusion from disinfectant spraying may cause damage to electric equipment and corrode sophisticated electronic components of the CT equipment. A previous study has reported that the machine should be shut down and covered with a plastic film before spraying disinfectants [19]; however, cross infection may occur when handling the plastic film, and repeated turning on and off of the machine also accelerates wear-out. Therefore, disinfectant spraying was not chosen in this experiment.

UV disinfection adopts UV-C rays within a wavelength range of 200–275 nm for irradiation, which has strong sterilization ability and performance stability [20]. Maintenance engineers have mentioned that the ozone released from UV irradiation may interfere with certain electronic components and may cause equipment damage and affect CT image quality, and frequent UV disinfection may accelerate aging of the casing and shorten the service life of the CT equipment; thus, our department had never previously installed a UV germicidal lamp in the CT room. Nevertheless, *Guidelines for the Diagnosis and Treatment of the Novel Coronavirus Pneumonia* (Trial edition 5) and numerous literature reports show a consensus that SARS-CoV-2 is sensitive to UV, resulting in robust inactivation. UV disinfection is thus one of the air disinfection methods that must be adopted for this COVID-19 epidemic. Under these circumstances, our department immediately installed UV germicidal lamps in various CT rooms and conducted this controlled experiment to evaluate its efficiency in disinfecting the CT room. UV germicidal irradiation must only be performed when nobody is around. However, patients come in randomly throughout the day. In addition, the disinfection effect decreases over time and with movement of personnel [21].

Air flow sterilization (natural ventilation or mechanical ventilation) is convenient and effective for air disinfection. The dust-free requirements of CT equipment make it impossible to design windows when setting up the CT room. Thus, plasma air sterilizers are commonly used in most CT rooms for air disinfection. Plasma air sterilizer is a combination of sterilization measures such as plasma, filtration, and electrostatic field [22]. The core of its function is the plasma reactor, which destroys the bacterial cell membrane under the action of a strong electric field and effectively kills microorganisms and aerosols [23]. Plasma air sterilizers have the advantage of high efficiency in sterilization, high degradability, low energy consumption, and long service life. Because of its easy operation, plasma air sterilizers can be operated when people are around and are not affected by the movement of people. It performs air disinfection continuously and dynamically, thus making up for the shortcomings of UV germicidal irradiation and thereby achieving better disinfection requirements.

The results of this study show that the difference in the number of bacterial colonies of the three disinfection methods at each time point after disinfection is statistically significant compared to that before disinfection (*p* < 0.01), indicating that the ultraviolet disinfection lamps, plasma air circulation disinfection machine, and ultraviolet disinfection lamp plus plasma air circulation disinfection machine impart good disinfection effects on the special CT machine room. According to the comparison among groups, the disinfection effect of the ultraviolet disinfection lamps combined with plasma air circulating air disinfector (group C) was better than that of ultraviolet disinfection lamps (group A) and plasma air circulating air disinfector (group B) (*p* < 0.05). Therefore, based on the persistence of disinfection effect and the sensitivity of the SARS-CoV-2 to UV, we recommend using UV germicidal irradiation combined with plasma air sterilization for air disinfection in the CT room that is specifically designated for COVID-19 during outbreaks.

It should be noted that strict quality control should be conducted in accordance with the disinfection principles recommended in the prevention and control plan. (1) At any time and fixed time, to achieve the continuous air disinfection effect, the plasma air disinfector should be used continuously with an average circulating air volume of ≥1,000 m^3^/h during the inspection period. At the end of the inspection, when no one is walking around, the ultraviolet disinfection lamps are turned on for disinfection for 60 min at the same time, three times a day. (2) Combined multimode disinfection: in view of the characteristics of SARS-CoV-2, the use of air disinfection alone is insufficient in disinfecting the area. Disinfection of the CT machinery and equipment, object surfaces, and floors should also be combined to achieve effective disinfection [24]; additionally, we should complete disinfection for CT examination couch which was touched directly by patients. In latest literatures, copper alloy was recommended because it could destruct the load property of microbe on its cover; therefore, copper alloy nano-coating could be combined with other disinfection methods in patient direct touch areas for optimistic effects [25]. (3) Use and maintenance of equipment: only the UV germicidal lamps that comply with national standards should be employed in air disinfection of CT rooms according to the *Technical Specifications for Disinfection of Medical Institutions* and actual needs. When installing the UV germicidal lamp tubes, they should be hanged beneath the lamp frame and covered with a polished aluminum plate reflector to ensure effectiveness of the disinfection. The lamp tubes should be wipe-cleaned with 75% alcohol every week. Attention should be paid to circuit protection when applying a plasma air sterilizer for uninterrupted dynamic sterilization. The sterilizer can be temporarily turned off during UV disinfection. The air outlet flaps and surface of the sterilizer should be wipe-cleaned everyday with disinfectant wipes containing quaternary ammonium compounds [26]. (4) Personnel control: although a complete disinfection method will be adopted when following the above guidelines, the persons entering the CT rooms should still be controlled. Healthcare professionals should put on secondary protection before entering the CT rooms. In addition, patients without mobility limitations should not be accompanied by family members and enter alone to avoid cross infection. (5) Monitoring and recording of the disinfection: management accountability should be implemented by organizing and compiling a registration form for the disinfection of CT rooms designated for COVID-19. The serial number, initial operation time, the start and end time of each irradiation, and cumulative irradiation duration of the UV germicidal lamp should be recorded. In addition, the time of usage, duration, and wipe-cleaning time of the plasma air sterilizers should also be recorded. Regular detection of the number of airborne bacterial colonies should be performed to ensure a satisfactory effect of the disinfection.

## 5. Limitation

Although we have made full preparations before the experiment, this study has several limitations. First, more samples should be collected for robust result and improving statistic power. Second, the disinfection method in this study was generally used in clinical settings; more new disinfection methods might be compared for more meaningful effects.

## 6. Conclusions

In summary, to fully respond to the COVID-19 epidemic and ensure the persistence of disinfection effect and safety of the CT rooms designated for COVID-19, we recommend the use of ultraviolet disinfection lamps combined with plasma air disinfection machine to disinfect the air in the special CT machine room for pneumonia, which can impart disinfection effects of continuous circulation, maximize the elimination of cross infection, and ensure the safety of doctors and patients. At the same time, it is suggested that in the process of implementation, it is necessary to establish a disinfection system in accordance with the norms and requirements based on the principle of protecting equipment and personnel and to conduct quality control management of compliance and effectiveness of disinfection.

## Figures and Tables

**Table 1:** Comparison of the number of air colonies before and after three disinfection methods (CFU/m)^3^.

Disinfection method	Before disinfection	Immediately after disinfection	2 h after disinfection
Group A	120.00 ± 45.54	21.58 ± 12.35	121.25 ± 56.63
Group B	120.02 ± 42.31	20.43 ± 8.69	97.10 ± 47.19
Group C	121.00 ± 28.98	21.10 ± 14.75	66.60 ± 41.66

**Table 2:** Comparison of the number of air colonies at different time points using three disinfection methods.

Time point	A vs. B		A vs. C		B vs. C	
	*t*	*p*	*t*	*p*	*t*	*p*
Immediately after disinfection	0.336	0.739	0.112	0.911	-0.183	0.856
2 h after disinfection	1.465	0.151	10.312^∗∗∗^	0.000^∗∗∗^	2.167	0.037^∗^

## Data Availability

The data used to support the findings of this study are available from the corresponding author upon request.
